# *StRAB4* gene is required for filamentous growth, conidial development, and pathogenicity in *Setosphaeria turcica*

**DOI:** 10.3389/fmicb.2023.1302081

**Published:** 2024-01-08

**Authors:** Pan Li, Hang Zhu, Chengze Wang, Fanli Zeng, Jingzhe Jia, Shang Feng, Xinpeng Han, Shen Shen, Yanhui Wang, Zhimin Hao, Jingao Dong

**Affiliations:** ^1^State Key Laboratory of North China Crop Improvement, Hebei Agricultural University, Baoding, China; ^2^College of Plant Protection, Hebei Agricultural University, Baoding, China; ^3^Hebei Bioinformatic Utilization and Technological Innovation Center for Agricultural Microbes, Hebei Key Laboratory of Plant Physiology and Molecular Pathology, College of Life Sciences, Hebei Agricultural University, Baoding, China

**Keywords:** *Setosphaeria turcica*, *StRAB4* gene, gene silencing, conidia, infection ability, RNA sequencing

## Abstract

*Setosphaeria turcica*, the fungal pathogen responsible for northern corn leaf blight in maize, forms specialized infectious structures called appressoria that are critical for fungal penetration of maize epidermal cells. The Rab family of proteins play a crucial role in the growth, development, and pathogenesis of many eukaryotic species. Rab4, in particular, is a key regulator of endocytosis and vesicle trafficking, essential for filamentous growth and successful infection by other fungal pathogens. In this study, we silenced *StRAB4* in *S. turcica* to gain a better understanding the function of Rab4 in this plant pathogen. Phenotypically, the mutants exhibited a reduced growth rate, a significant decline in conidia production, and an abnormal conidial morphology. These phenotypes indicate that StRab4 plays an instrumental role in regulating mycelial growth and conidial development in *S. turcica*. Further investigations revealed that StRab4 is a positive regulator of cell wall integrity and melanin secretion. Functional enrichment analysis of differentially expressed genes highlighted primary enrichments in peroxisome pathways, oxidoreductase and catalytic activities, membrane components, and cell wall organization processes. Collectively, our findings emphasize the significant role of StRab4 in *S. turcica* infection and pathogenicity in maize and provide valuable insights into fungal behavior and disease mechanisms.

## Introduction

Northern corn leaf blight (NCLB) is a highly destructive foliar disease that significantly impacts global maize production (Zeng et al., 2020). It is characterized by the emergence of elongated, grayish-greenish and elliptical lesions on maize leaves, which have detrimental effects on both grain yield and quality (Perkins and Pedercens, 1987; Van Inghelandt et al., 2012). The severity of NCLB varies depending on a multitude of factors, including the environmental conditions, the genetic makeup of the maize plant, and the virulence of the pathogen. These variables pose a significant challenge for maize growers and plant breeders working to develop resistance to NCLB.

The causative agent responsible for NCLB is the fungal pathogen *Setosphaeria turcica*. The pathogen primarily spreads through asexual conidia, which is the main mode of transmission. Upon landing and adhering to the maize leaf surface, the conidia germinate to form a germ tube that expands and forms an appressorium. The appressorium plays a crucial role in enabling the direct penetration of fungal hyphae into epidermal cells by forming a penetration peg, which utilizes the turgor pressure accumulated within the appressorium (Zeng et al., 2020). Once the epidermal cells are penetrated, the terminal Spitzenkörper (acrosome) undergoes polar growth, facilitating mycelial extension into deeper plant cell layers. Subsequently, materials are transported from the accumulated secretory vesicles to the growing tips at mycelial ends.

Rab proteins, small monomeric GTP-binding proteins with a molecular weight ranging from 21 to 25 kDa, constitute the largest subfamily of GTPases within the Ras superfamily (Stenmark and Olkkonen, 2001). These proteins are highly conserved across eukaryotic organisms and play pivotal roles in regulating processes such as endocytosis and exocytosis during vesicular transport (Stenmark, 2009; Mizuno-Yamasaki et al., 2012; Pfeffer and Kellogg, 2017). Rab proteins function as molecular switches, oscillating between active GTP binding states and inactive GDP binding states to facilitate vesicle budding, transport, docking, and fusion (Hutagalung and Novick, 2011; Pfeffer, 2013; Wandinger-Ness and Zerial, 2014; Li and Marlin, 2015). Emerging evidence highlights the significance of endocytosis in fungal development and pathogenicity, a process heavily dependent on vesicle transport orchestrated by the Rab family, which are universally conserved across various life kingdoms. Rab5 homologs have been demonstrated to promote early endosomal fusion in multiple fungal species (Ohya et al., 2009), with a genetic knockout of MoRab5 in *Magnaporthe grisea* leading to fungal growth impairments (Yang et al., 2017). Additionally, Rab family members in *Fusarium graminearum* are essential for the pathogenicity of fungi and the production of the mycotoxin deoxynivalenol (Zheng et al., 2015). Rab4 homologs in *Schizosaccharomyces pombe* and various filamentous fungi contribute to the recycling of membrane proteins (Van der Sluijs et al., 1992), resulting in altered cell wall permeability (Jones et al., 2006). These findings underscore the pivotal role of Rab proteins in vesicle trafficking, which is crucial for fungal development, polar growth, and pathogenicity.

Previous work conducted by our group has demonstrated that appressorium-mediated infection in *S. turcica* is governed by cyclic AMP and mitogen-activated protein kinase signaling pathways (Liu et al., 2022). It was revealed that StPkaC1 and StpkaC2, two PKA-C subunits functioning downstream of the cAMP signaling pathway, negatively regulate the transcription of *StRAB4* (formerly *StRAB1*), which encodes a Rab homolog in *S. turcica* (Liu et al., 2022). Given the significant impact of StPkaC2 in the filamentous growth of *S. turcica*, we aimed to explore the relationship between PKA and chitin biosynthesis (Liu et al., 2022). Our investigation revealed that *StRAB4* (JGI database ID 162018), a Rab-GTPase homolog co-localizing with chitin synthase at the mycelial tip in *Neurospora crassa* (Sánchez León et al., 2015), was significantly upregulated in both *ΔStPkaC2* and *ΔStPkaC1* mutants (Liu et al., 2022). These results suggest that StPkaC1 and StPkaC2 negatively regulate the transcription of chitin biosynthesis-related genes, particularly *StRAB4*. Notably, the Calcofluor white (CFW) staining pattern in the *StRAB4* RNAi mutant was notably faint, including at the hyphal septa, implying inactive chitin synthesis (Liu et al., 2022). While *ΔStPkaC1* also exhibited increased chitin content and upregulated chitin synthase expression, it did not manifest the same phenotype of accelerated filamentous growth as *ΔStPkaC2* (Liu et al., 2022). The differences in the upregulated levels of *StRAB4* in the two PKA-C subunit mutants strongly suggest that the functional distinctions between these subunits are primarily driven by their transcriptional regulation of *StRAB1*. This implies that StRab4 plays a pivotal role in the filamentous growth of *S. turcica* as a downstream effector within the cAMP signaling pathway.

This study aims to elucidate the role played by *StRAB4* in *S. turcica* pathogenicity. We have employed RNA interference (RNAi) technology to explore the functions of *StRAB4* in hyphal development and pathogenicity. Our findings reveal valuable insights into the relationships between *StRAB4* and fungal filamentous growth, cell wall integrity, conidiation, and appressorial development. This research enhances our understanding of the regulatory mechanisms involved in the pathogenicity of *S. turcica*, laying the foundational basis for the development of effective strategies to manage plant diseases.

## Materials and methods

### Strains, plant material, and culture conditions

The *S. turcica* wild-type (WT) strain 01-23 was isolated from maize leaves displaying symptoms of NCLB in Liaoning Province, China. The WT strain 01-23 was characterized and deposited under reference number 9857 in the China General Microbiological Culture Collection Center. Both the WT and WT-derived *StRAB4* RNAi strains (*StRab4i-1, StRab4i-2,* and *StRab4i-4*) were grown on potato dextrose agar (PDA) (20% potato infusion, 2% glucose, and 1.5% agar) at 25°C and periodically subcultured to maintain their viability. The maize inbred line B73, which served as the susceptible host for *S. turcica* infection, was cultivated in a growth chamber under long-day conditions (16 h of light, 8 h of darkness). The temperature inside the growth chamber was maintained at 25°C during the light period and 18°C during the dark period. Maize leaves inoculated with *S. turcica* were placed at a temperature of 25°C (Liu et al., 2022).

### Generation of *ΔStRAB4* mutants

Knockout mutants of *StRAB4* were generated through the utilization of homologous recombination. We have chosen a total of 500 base pairs (bp) of flanking sequences, both upstream and downstream of the coding region of *StRAB4*, to serve as homology arms I and II. Subsequently, primers were constructed for the purpose of cloning homology arms I and II. The homologous arm that underwent cloning was subjected to purification and subsequently linked to the pBS-bar vector. Recombinant pBS-I-bar-II plasmids were introduced into protoplasts of WT strain by polyethylene glycol (PEG)-mediated transformation. Targeted replacement of *StRAB4* was performed using bialaphos (bar) markers to generate *ΔStRAB4* mutants. The *StRAB4*:bar constructs were introduced into *S. turcica*, and transformants were selected on PDA containing 200 μg/mL glufosinate. Vector construction for allelic replacement to generate the *ΔStRAB4* mutant was performed as described previously (Liu et al., 2022). All primers used are listed in Supplementary Table S1.

### Generation of *StRAB4* RNAi mutants

*StRAB4* RNAi constructs were expressed using the pSilent-1 vector, following standard procedures with modifications based on the targeted sequences for *StRAB4*, as described previously (Liu et al., 2022). Specifically, two distinct target sites within the *StRAB4* mRNA sequence were selected to design the hairpin sequences for *StRAB4* silencing. The amplified fragments were then inserted into the pSilent-1 vector to generate the *StRAB4*-silencing constructs, which we designated as pSilent-Bar-*StRAB4* (Supplementary Figure S1). All primers used in this study are provided in Supplementary Table S1. These constructs were transformed into protoplasts derived from the WT using a protocol described in our previous study (Zhang et al., 2012). Transformants were screened on PDA containing Basta (Coolaber) and confirmed through quantitative polymerase chain reaction (qPCR) to detect the *Bar* gene.

### RNA-Seq experimental design and data analysis

RNA-sequencing and data analysis were performed as described previously (Meng et al., 2022). Sequencing was performed using the Illumina HiSeq 4000 platform. Raw data underwent a filtering process to remove adaptor sequences and low-quality reads. High-quality paired reads were then aligned to the reference genome of *S. turcica* strain Et28A (GenBank accession: CP054627 to CP054656, PRJNA638013). Cufflinks was used to calculate fragments per kilobase of transcript per million mapped reads (FPKM) values for individual genes, and the log2 ratio of FPKM values between the WT and *StRab4i-2* sample libraries was used to evaluate differences in expression. Each sample consisted of three biological replicates. The RNA-Seq data are available under accession number SUB13852965 on the NCBI server.^1^ Differentially expressed genes (DEGs) were identified using a false discovery rate (FDR)-corrected *p*-value of ≤0.01. Gene annotation was performed by BLASTing against a local Nr database.^2^ Furthermore, Clusters of Orthologous Groups (COG; http://www.ncbi.nlm.nih.gov/COG/), Gene Ontology (GO; http://www.geneontology.org/), and Kyoto Encyclopedia of Genes and Genomes (KEGG; http://www.genome.jp/kegg/) databases were used to assign gene functions (Meng et al., 2023; Zhang et al., 2023).

### RNA extraction and real-time quantitative PCR analysis of gene expression

Total RNA was extracted from *S. turcica* using an E.Z.N.A.^®^ Fungal DNA Mini Kit (Omega Bio-Tek). Subsequently, the extracted RNA was reverse-transcribed to complementary DNA (cDNA) using the ABScript II Reverse Transcriptase (ABclonal), according to the manufacturer’s instructions. Gene expression levels were quantified by qPCR analysis using the 2X Universal SYBR Green Fast qPCR Mix (ABclonal) on the CFX96 Real-Time System (BioRad) as described previously (Meng et al., 2022). To estimate the expression levels of the target genes, the data were processed using the 2^−ΔΔCT^ method, with the expression of the β-tubulin gene serving as the internal control (Bustin et al., 2009; Zeng et al., 2020).

### Phenotypic analyses

The WT and RNAi strains were inoculated on PDA plates and incubated in the dark at 25°C for 7 days, after which the morphology of each colony was assessed. To quantify colony growth rates for both the WT and RNAi strains, the strains were cultivated on PDA plates at 25°C in the dark, with measurements of colony diameter taken at 24 h intervals over a 7 days period (Zeng et al., 2020). Each experiment was conducted in triplicate, with measurements collected from 10 Petri dishes for each biological replicate.

### Monospore isolation and subculture

Conidia from the mutant strains were collected, diluted to a concentration of 100 conidia/mL, and evenly spread onto water agar plates to air dry. Once dried, individual conidia with abnormal morphology were identified under a light microscope and labeled. Conidia that successfully germinated were selected and inoculated onto PDA plates, followed by incubation overnight at 25°C (Shen et al., 2013). Subsequently, these conidia were transferred to new PDA plates and stored for further studies. Four abnormal conidia were isolated from the *StRab4i-2* strain. These conidia were cultured separately and named *StRab4i-S1*, *StRab4i-S2*, *StRab4i-S3*, and *StRab4i-S*. These strains were cultured on PDA plates and incubated at 25°C for 10 days. Colonies from both the WT and *StRab4i-S* strains were inoculated, cultured for an additional 10 days, and then imaged.

### Cell wall integrity analysis and surface hydrophobicity determination

The WT and *StRab4i* strains were individually inoculated on three PDA plates containing 100 μg/mL Congo Red, 20 μg/mL CFW, and 0.01% sodium dodecyl sulfate (SDS). Colony diameters were measured, and inhibition rates were calculated using the formula: Inhibition rate (%) = (1 − administered colony diameter/CK colony diameter) × 100% (Ram and Klis, 2006).

To observe wettable areas, the WT and *StRab4i* colonies were grown for 10 days before 30 μL deionized water (ddH_2_O), 0.2% gelatin, 250 mg/mL Tween-20, and 0.2% SDS with 5 mM ethylenediaminetetraacetic acid were added. Subsequently, the changes in colony wettability were assessed.

### Microscopic observation of conidial yield

Mycelia from both the WT and RNAi strains were inoculated on PDA and incubated for 10 days at 25°C. Following the incubation period, mycelia were collected from each Petri dish using 5 mL ddH_2_O. To isolate hyphae of the same age, two layers of gauze were used. Subsequently, 10 μL the flow-through containing the conidia was spotted on the surface of a coverslip. The number of conidia was counted using an Eclipse E-200 microscope (Nikon) as described previously (Liu et al., 2022). Each experiment was repeated three times, and for each repetition, conidial counts were taken from 10 Petri dishes.

### Appressorium formation and penetration assays

Vegetative hyphae from both the WT and RNAi strains were harvested from 10 days-old PDA cultures and suspended in sterile water. Hyphal suspensions were then applied to an artificial cellophane surface and incubated at 25°C. Appressorium formation and mycelial penetration of the cellophane surface were assessed under a light microscope at 6, 12, and 24 h post-inoculation. Simultaneously, WT and RNAi strains were inoculated on a PDA plate covered with an artificial cellophane surface. The plates were then incubated in an inverted position at 25°C for a period of 5 days and then photos were taken. Following the incubation period, the artificial cellophane surface was removed, and the strains were further cultured at 25°C for an additional 5 days and then photos were taken (Gu et al., 2014; Liu et al., 2022).

### Plant infection assays and melanin extraction

Maize leaf lesions were examined using a plant infection assay as described previously with minor modifications (Ma et al., 2017). Droplets containing 100 μL of a conidial suspension (1 × 10^6^ conidia/mL), with or without the specified reagent, were applied to leaves of the inbred maize line B73 before placing the inoculated leaves in a growth chamber under long-day conditions at 25°C (Zeng et al., 2020). Disease spots formed by early infection that appeared after 2 days were sampled, imaged, and counted. Data were obtained from three independent replicates.

Intracellular melanin extraction was performed using 0.1 g dried mycelia from the specified strains, and quantification was conducted using a previously described method with minor modifications (Zeng et al., 2020). Conidia collected from the strains were cultured in flasks containing 100 mL potato dextrose broth (PDB; 20% potato infusion, 2% glucose) at 25°C with shaking at 110 rpm for 24 h. The strains were then cultured for an additional 4 days. The samples were boiled for 5 min and centrifuged at 13,400 g for 10 min, and the resulting mycelial pellet was washed and dried. Approximately 0.1 g the dried mycelia was used for melanin extraction.

Extracellular melanin was extracted by collecting the sporulation suspension and transferring it into an Erlenmeyer flask containing 100 mL PDB, followed by incubation at 25°C with or without shaking at 110 rpm for 10 days. The mycelia were pressed dry with filter paper and weighed. The culture medium was filtered through a double-layered sterile filter paper, and the pH of the filtrate was adjusted to 2 with 7 M HCl. The mixture was incubated overnight at 4°C to precipitate melanin, followed by centrifugation at 13,400 g for 10 min. The supernatant was discarded, and the precipitate was washed with 1 mL ddH_2_O and then dissolved in 1 mL 1 M NaOH for 2 h at room temperature. The extract was centrifuged again at 13,400 g for 10 min, and the supernatant was removed and saved. Melanin was precipitated from the supernatant with acid and then dissolved with alkali. The color of the melanin precipitate was observed and imaged, and the absorption of the precipitate at 400 nm was measured with a spectrophotometer (Zeng et al., 2020).

## Results

### *StRAB4* regulates filamentous growth and conidiation in *Setosphaeria turcica*

**Figure 1:**
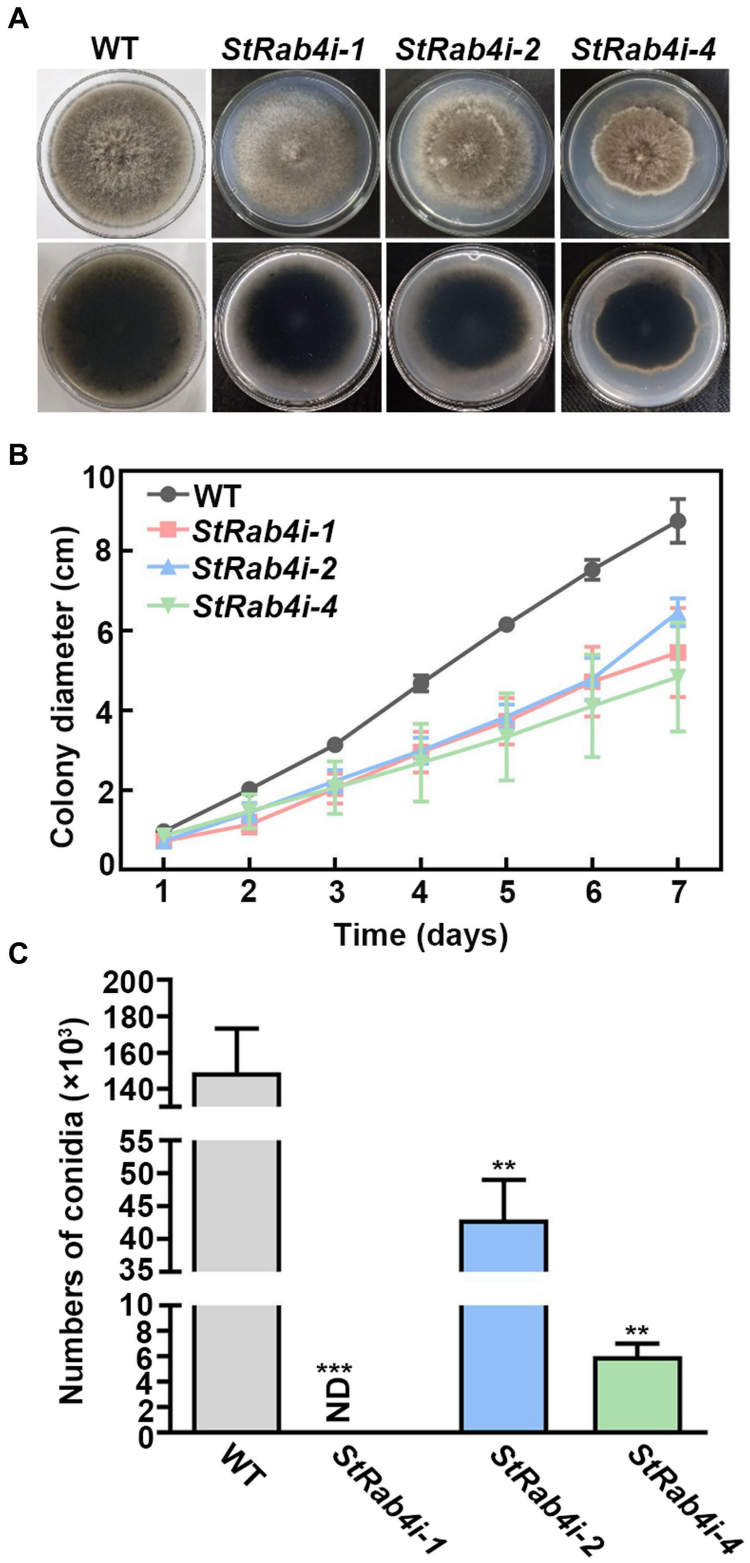
StRab4 is required for *S. turcica* hyphal development and is essential for maize leaf infection. **(A)** Images of WT and *StRab4i* strains growing on PDA plates at 25°C. **(B)** Growth curve of WT and *StRab4i* strains growing on PDA plates over the course of 7 days. Error bars represent the standard error of three biological replicates. **(C)** Conidial production of WT and *StRab4i* strains. Bar chart shows the numbers of conidia produced by WT and *StRab4i* strains. ND indicates not detected. Asterisks indicate statistical significance, as determined by two-tailed student’s *t*-test with three independent experimental replicates (^**^p < 0.01 and ^***^p < 0.001). Error bars represent the standard deviation of three replicates.

We failed in our attempt to produce a *StRAB4* knockout mutant. This might be because Rab belongs to the Ras family in which some members are fatal following knockout (Stenmark and Olkkonen, 2001; Park et al., 2006). Alternatively, it could be because *S. turcica* is a polykaryotic fungus, which makes it challenging to obtain knockout mutants. Therefore, we silenced *StRAB4* in *S. turcica* WT strain 01-23 using RNAi to investigate its role in fungal development and pathogenicity. Silencing was confirmed in three strains (*StRab4i-1*, *-2*, and *-4*) through both RT-qPCR and qPCR (Supplementary Figure S1). In terms of morphology and growth, the WT displayed a characteristic appearance with a dark brown, dense, and fluffy mycelial mat, consisting of slender individual mycelia that extended to the border of the petri dish after 7 days of growth. Conversely, the *StRab4i-1* strain exhibited grayish-whitish colony growth with sparser mycelia. The *StRab4i-2* and *StRab4i-4* strains developed grayish-brownish colonies, sparse mycelia, and irregular growth patterns (Figure 1A). During the initial 6 days of cultivation on PDA plates, the growth rates of the *StRab4i-1* and *StRab4i-2* strains were relatively similar, although the *StRab4i-2* strain exhibited slower growth (Figure 1B). Additionally, the RNAi strains exhibited significantly reduced sporulation compared to the WT, with the *StRab4i-1* strain failing to sporulate altogether (Figure 1C). In summary, all *StRab4i* strains exhibited lighter colony colors, irregular colony morphologies, and slower growth patterns compared to the WT, suggesting *StRAB4* influences mycelial growth, conidial development, and pigment production.

### *StRAB4* silent strain resulted in abnormal conidial morphology and weakened pathogenicity

**Figure 2:**
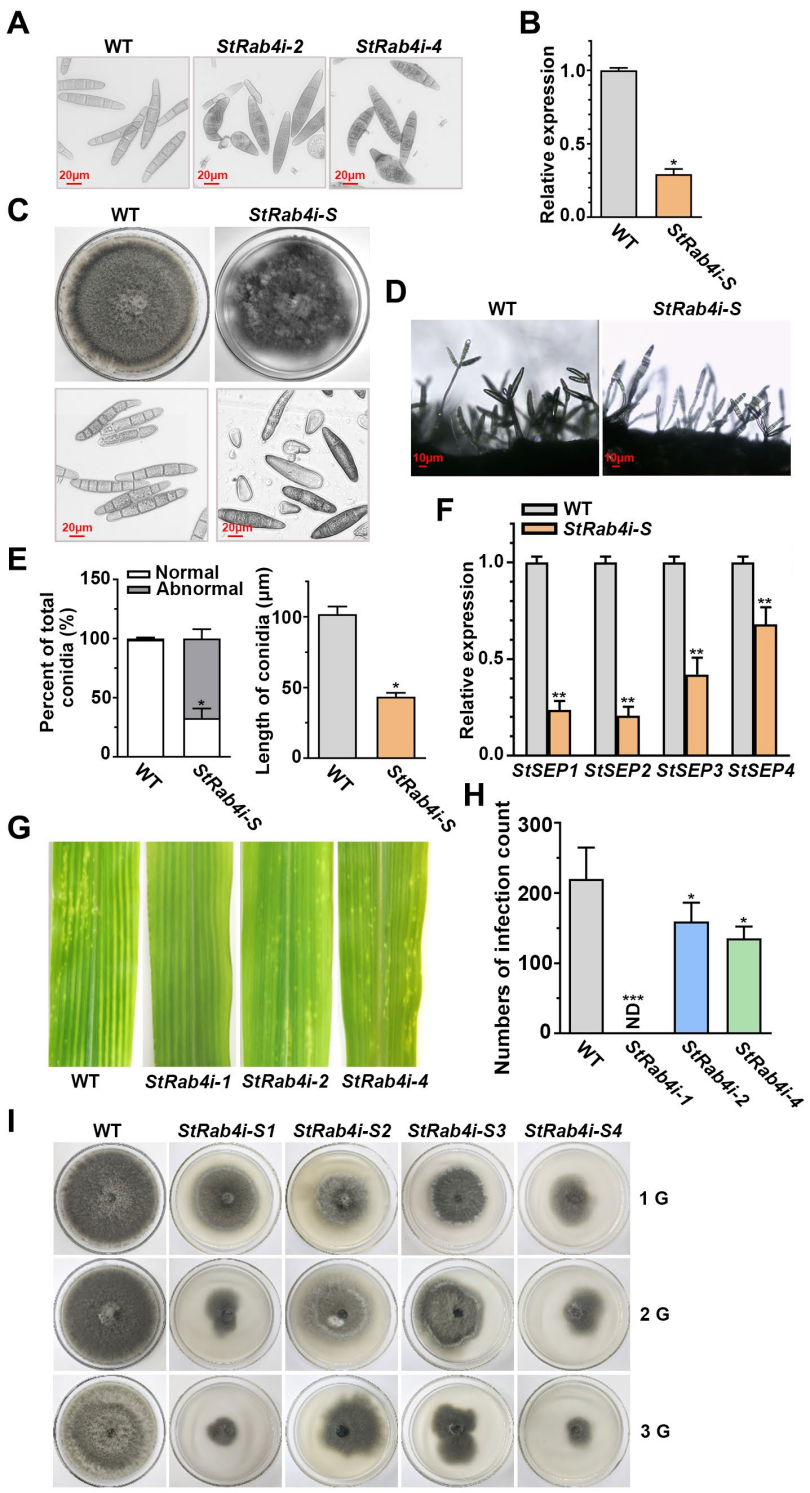
StRab4 regulates conidial and mycelial morphology. **(A)** Conidial morphology of WT and *StRab4i* strains as observed under a microscope (scale bar, 20 μm). **(B)** *StRAB4* expression in the *StRab4i-S* strain. Asterisks indicate statistical significance, as determined by two-tailed student’s *t*-test (^*^p < 0.05). Error bars represent the standard deviation (SD) of three replicates. **(C)** Colony morphology of the WT and *StRab4i-S* strains on PDA plates after 7 days of growth at 25°C and microscopic views of conidia (scale bar, 20 μm). **(D)** Conidiophore morphology of WT and *StRab4i-S* strains (scale bar, 10 μm). **(E)** Bar chart showing the percentage of conidia with WT-like (normal) and abnormal morphology produced by the WT and *StRab4i-S* strains (left) and the length of conidia from the WT and *StRab4i-S* strains (right). Asterisks indicate statistical significance, as determined by two-tailed student’s *t*-test (^*^p < 0.05). Error bars represent SD of three replicates. **(F)** Reverse transcription-quantitative PCR (RT-qPCR) analysis of the relative expression of septin genes in the WT and *StRab4i-S*. Asterisks indicate statistical significance, as determined by two-tailed student’s *t*-test (^**^p < 0.01). Error bars represent the SD of three replicates. **(G)** Early infection counts of maize leaves infected with conidia from the WT and *StRab4i* strains at 2 days post-inoculation. **(H)** Quantification of early infection counts caused by the WT and *StRab4i* strains. ND indicates not detected. Asterisks indicate statistical significance, as determined by two-tailed student’s *t*-test (^*^p < 0.05 and ^***^p < 0.001). Error bars represent the SD of three replicates. **(I)** WT and *StRab4i-S* strains were subcultured on PDA plates for multiple generations: first (1G), second (2G), and third (3G) generations.

As the *StRab4i-1* strain did not produce conidia, we collected conidia from the WT, *StRab4i-2*, and *StRab4i-4* strains only (Figure 2A). The WT conidia were brown and spindle-shaped, with most containing between four and seven septa. Conversely, while most conidia from the *StRab4i-2* and *StRab4i-4* strains appeared normal, some lacked color or displayed uneven color patterns, reduced septa, and irregular morphologies. Some conidia had one enlarged end, between one and three septa, and an uneven color pattern, while others were short and colorless, lacked a septum, and featured a ruptured end with a light color. Taken together, these observations suggest the *StRAB4* gene plays a role in regulating *S. turcica* conidia morphogenesis.

Abnormal conidia were isolated from the *StRab4i* mutants, and it was observed that the short, colorless conidia without septa could not germinate, whereas the abnormal colored conidia with septa could germinate. A germinating conidium with abnormal morphology was isolated to generate the *StRab4i-S* strain. In the *StRab4i-S* strain, the expression of the *StRAB4* gene was approximately 70% lower than in the WT and lower than in the original RNAi strain (Figure 2B). The *StRab4i-S* strain grew significantly slower than the WT on PDA and exhibited an extremely irregular colony morphology (Figure 2C). Additionally, abnormalities were observed in the conidia and conidiophores of the *StRab4i-S* strain (Figures 2C,D). There was only one conidium on the majority of the mutant’s conidiophores, while 2–3 conidia are borne on most conidiophores of the WT. Abnormal conidia collected from the *StRab4i-S* strain could differentiate into normal conidia, but the proportion was relatively low (Figure 2E). Most conidia retained different aspects of abnormal morphology. The analysis revealed that the abnormal rate of conidia morphology in the *StRab4i-S* strain reached 67.03%, which was more than two-thirds higher than the original mutant strain without single-conidium isolation (Figure 2E). The average conidial length of the *StRab4i-S* strain was slightly less than half that of the WT (Figure 2E). A previous study has demonstrated that septin proteins of pathogenic fungi are involved in cell polarity and morphology, as well as morphological changes related to pathogenesis (Ryder et al., 2019). To understand the underlying mechanisms, we performed relative quantitative analysis of four septin genes in the *StRab4i-S* strain (Figure 2F). The results showed that the relative expression of the four septin genes in the *StRab4i-S* strain was significantly lower than that in the WT, suggesting *StRAB4* gene regulates the formation of the conidial diaphragm by influencing the expression of septin genes.

We have previously investigated the pathogenicity of inoculated mycelia and found that the mycelia of *StRab4i-1* did not cause any lesions, whereas both *StRab4i-2* and *StRab4i-4* could cause lesions (Liu et al., 2022). In order to further explore whether conidium can cause maize leaf disease, the pathogenicity of the *StRab4i* RNAi strains was assessed by inoculating equal numbers of conidia onto the center of maize leaves. Two days post-inoculation, lesions could be seen on leaves inoculated with the WT, *StRab4i-2*, and *StRab4i-4* strains (Figure 2G). However, fewer lesions developed on leaves inoculated with the *StRab4i-2* and *StRab4i-4* strains compared to those inoculated with the WT (Figure 2H). As expected, leaves inoculated with the *StRab4i-1* strain did not develop any lesions because of the inability of this strain to produce conidia.

In the subculture experiment involving WT and *StRab4i-S* strains, it was observed that after 10 days of growth, the first-generation *StRab4i-S* strain showed normal colony morphology and slower growth compared to the WT (Figure 2I). The second-generation *StRab4i-S* strain exhibited abnormal colony morphology and slower growth after 10 days, with some colonies having a diameter less than 4 cm. After 10 days of growth, the third-generation *StRab4i-S* strain exhibited extremely abnormal colony morphology, with some colonies having a diameter less than 4 cm and others barely growing. The *StRab4i-S* strain could not be subcultured, which indicated particularly weak survival. This experiment reflects the influence of abnormal spore morphology on the growth and development of colonies, as evident by their inability to be successfully subcultured, and highlights the importance of the *StRAB4* gene in the growth and development of *S. turcica*.

### *StRAB4* silent strain regulates the development of infection structures

**Figure 3:**
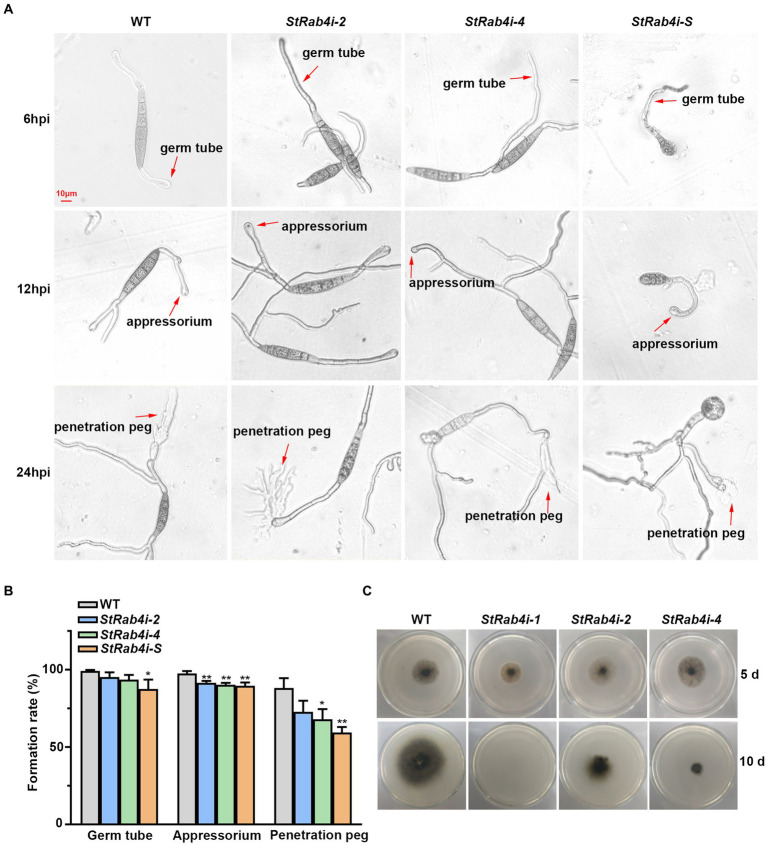
StRab4 regulates conidial germination and hyphal penetration. **(A)** Micrographs depicting germ tube, appressorium, and penetration peg formation from individual conidia of the WT and *StRab4i* strains growing on a cellophane layer over PDA. Images were taken at 6, 12, and 24 h post-inoculation (hpi) (scale bar, 10 μm). **(B)** Quantification of germ tubes, appressoria, and penetration pegs from conidia of WT and *StRab4i* strains growing on a cellophane layer over PDA. Asterisks indicate statistical significance, as determined by two-tailed student’s *t*-test with three independent replicates (^*^p < 0.05 and ^**^p < 0.01). Error bars represent the standard deviation (SD) of three replicates. **(C)** Images depicting penetration by WT and *StRab4i* strains growing on a cellophane layer over PDA. Images were taken at 5 and 10 days post-inoculation.

Cellophane mimics the hydrophobic surface of maize leaves and serves as a good *in vitro* model for assessing *S. turcica* penetration through epidermal cells (Zeng et al., 2020; Liu Y. et al., 2021). We placed a cellophane layer over water agar and inoculated the surface with conidia from the WT, *StRab4i-2*, *StRab4i-4*, and *StRab4i-S* strains. Six hours post-inoculation, the WT conidia germinated and formed germ tubes at both ends, while those of *StRab4i* strains germinated at one end only, with abnormal conidia exclusively doing so (Figure 3A). By 12 h, the WT conidia typically formed appressoria at both ends, while those of *StRab4i* strains formed one or none (Figure 3A). After 24 h, the WT conidia generated invasive hyphae at one or both ends. Conversely, the conidia of the *StRab4i* strains produced invasive hyphae at one end only, with some abnormal conidia failing to produce invasive hyphae but exhibiting small, branched structures at one end only (Figure 3A). Additionally, during the entire infection process, the sprout tubes of normal spores of the *StRab4i* strains were longer than those of the WT, and the more the sprout tubes of the abnormal spores grew, the more chaotic they became in their morphology and appearance (Figure 3A).

We calculated the germination, appressorium formation, and penetration peg formation rates of the WT and *StRab4i* strains to quantify our observations. The germination and appressorium formation rates were consistently, but only slightly, lower in the *StRab4i* strains than in the WT (Figure 3B). The reduction in the penetration peg formation rate in the *StRab4i* strains was more obvious. Compared to the WT, the penetration peg formation rate was reduced by approximately 15, 20, and 29% in the *StRab4i-2*, *StRab4i-4*, and *StRab4i-S* strains, respectively (Figure 3B). The WT and *StRab4i* mutants were inoculated on a cellophane layer covering a PDA plate to assess whether the reduced penetration peg formation observed in the *StRab4i* strains correlated with a reduced penetration ability. The *StRab4i-2* and *-4* strains, while capable of penetrating the cellophane layer, exhibited significantly reduced penetration success compared to the WT (Figure 3C). Conversely, the *StRab4i-1* strain was unable to penetrate the cellophane layer because of its inability to produce conidia (Figure 3C). Taken together, these findings indicate that *StRAB4* plays a crucial regulatory role in the formation of *S. turcica* infection structures, which is essential for the pathogenicity of the fungus.

### *StRAB4* regulates cell wall integrity

The fungal cell wall is a critical structural component primarily composed of chitin, glucan, chitosan, and cellulose, and it plays a vital role in maintaining cell morphology and integrity (Lu et al., 2023). Congo Red, a fungal cell wall inhibitor, specifically binds to β-1,3-glucans, disrupting cell wall assembly and inhibiting fungal growth (Ram and Klis, 2006; Liu Z. et al., 2021). CFW interferes with cell wall assembly by binding to chitin monomers and preventing their polymerization (Ram and Klis, 2006). SDS, an ionic detergent, disrupts cell membranes (Zhao et al., 2020). These compounds are valuable tools for evaluating the cell wall integrity of fungal mutants, as defects in cell wall integrity typically result in increased sensitivity to these compounds.

**Figure 4:**
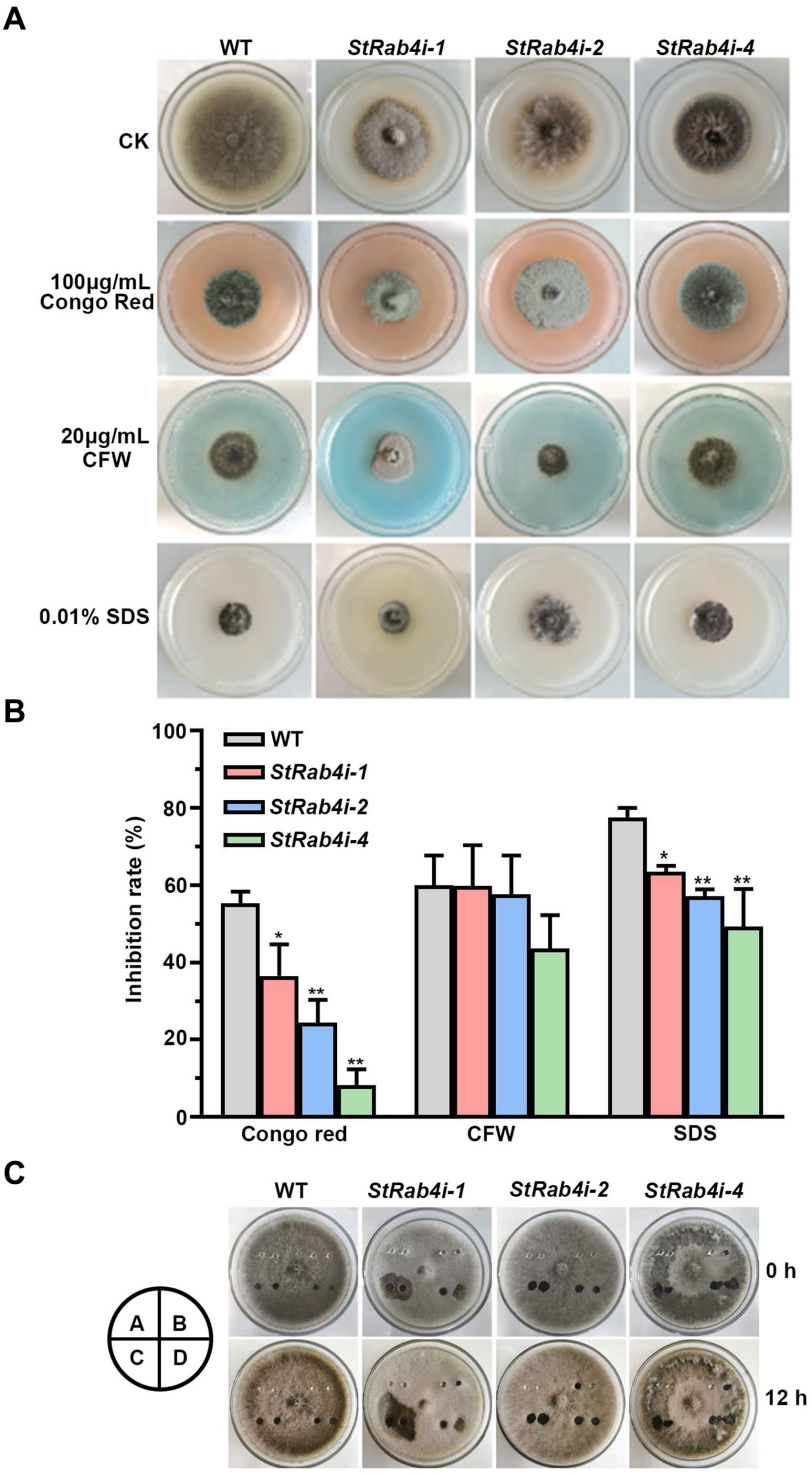
StRab4 regulates cell wall integrity and surface hydrophobicity in *S. turcica*. **(A)** WT and *StRab4i* strains were inoculated on PDA plates containing 100 μg/mL Congo Red, 20 μg/mL calcofluor white (CFW), 0.01% sodium dodecyl sulfate (SDS), and control (CK) plates containing no additional chemicals. **(B)** Inhibition rate of WT and *StRab4i* strains growing on PDA plates containing 100 μg/mL Congo Red, 20 μg/mL CFW, and 0.01% SDS was quantified by normalizing to growth on the CK negative control plate. Asterisks indicate statistical significance, as determined by two-tailed student’s *t*-test with three independent replicates (^*^p < 0.05 and ^**^p < 0.01). Error bars represent the standard deviation of three replicates. **(C)** Determination of surface hydrophobicity in WT and *StRab4i* strains. Colonies were treated with a 30 μL H_2_O, 0.2% gelatin, 250 mg/mL Tween-20, or 0.2% SDS with 5 mM EDTA. Images were taken at 0 and 12 h post-inoculation (hpi). A: H_2_O; B: 0.2% gelatin; C: 250 mg/mL Tween 20; D: 0.2% SDS with 5 mM EDTA.

We treated the WT and *StRab4i* strains with 100 μg/mL Congo Red, 20 μg/mL CFW, and 0.01% SDS. Congo Red, CFW, and SDS clearly inhibited the growth of both the WT and *StRab4i* strains (Figures 4A,B). However, all *StRab4i* strains displayed lower sensitivity to Congo Red and SDS compared to the WT (Figures 4A,B). While CFW treatment had a similar effect on the WT, *StRab4i-1,* and *StRab4i-2* strains, the *StRab4i-4* strain exhibited slightly lower sensitivity (Figure 4B), suggesting the *StRab4* gene positively regulates cell wall integrity in *S. turcica*.

Fungal surface hydrophobicity is involved in several aspects of fungal growth and development. It is also a surface property influencing microbial interactions at the fungal interface (Chau et al., 2009). The surface hydrophobicity of the WT and *StRab4i* strains were evaluated by treating the surface of growing colonies with 30 μL H_2_O, 0.2% gelatin, 250 mg/mL Tween-20, and 0.2% SDS with 5 mM EDTA and assessing if these solutions spread over the surface of the colony or remain pooled in a droplet (Figure 4C). Water did not spread when added to the surface of WT or *StRab4i* strains after 12 h. Gelatin did not spread on the surface of the WT but spread slightly on the surface of the *StRab4i* strains. Tween-20 spread slightly on the surface of the WT but spread substantially on the surface of the *StRab4i* strains. SDS spread on both the WT and *StRab4i* strains but spread more on the *StRab4i* strains. These results indicate that the surface hydrophobicity of the *StRab4i* strains is lower than that of the WT, as gelatin, Tween-20, and SDS exhibited greater spreading.

### *StRAB4* plays an important role in the synthesis of melanin

**Figure 5:**
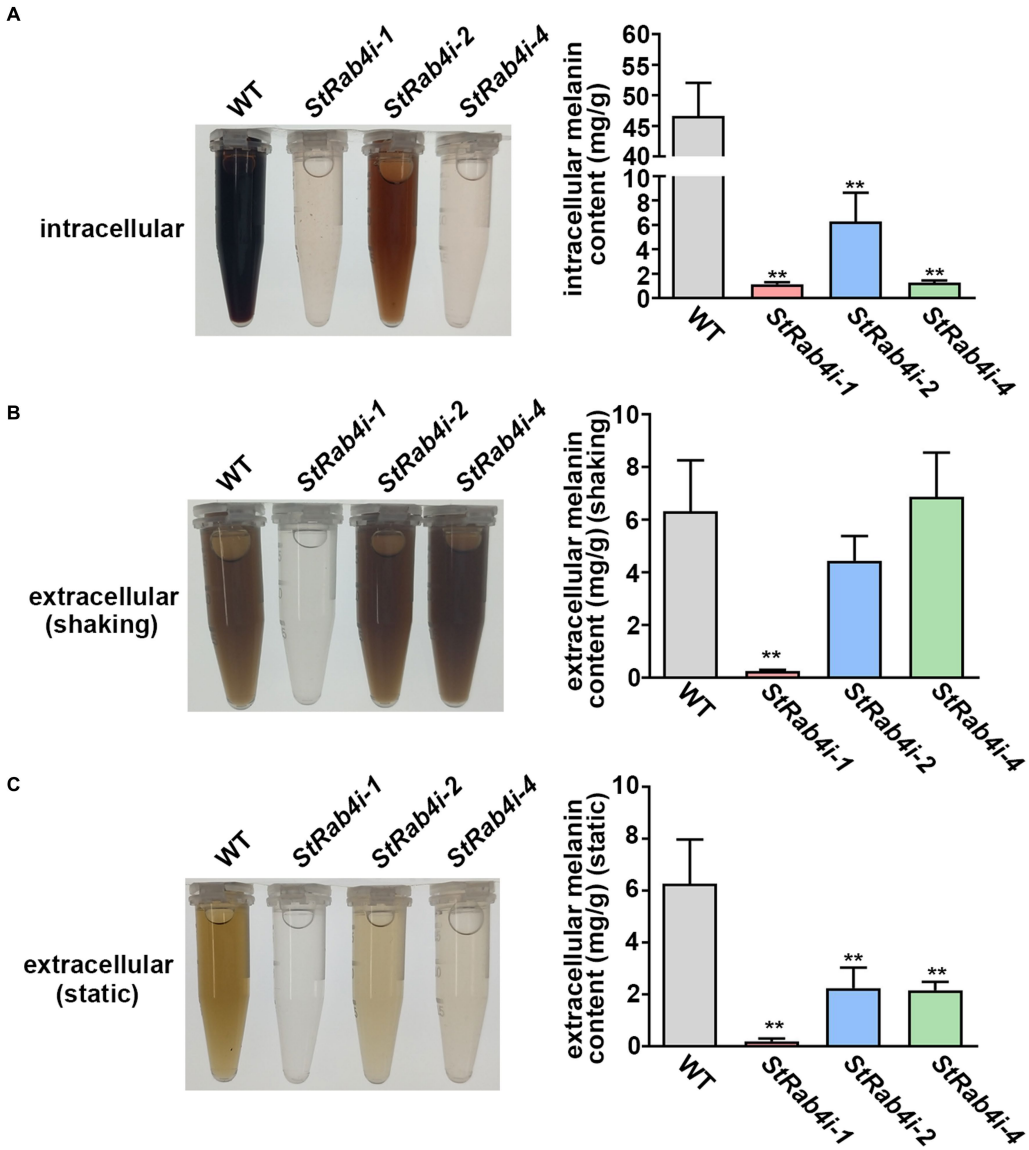
StRab4 regulates melanin synthesis in *S. turcica*. Intracellular melanin was extracted from WT and *StRab4i* strains in liquid culture with shaking. Extracellular melanin was extracted from fungal strains growing in liquid culture in the presence (shaking) and absence (static) of shaking. **(A)** Intracellular melanin in fungal extracts was black-to-light brown in color depending on the concentration and intracellular melanin content from WT and *StRab4i* cultures was quantified. Asterisks indicate statistical significance, as determined by two-tailed student’s *t*-test with three independent replicates (^**^p < 0.01). Error bars represent the standard deviation of three replicates. **(B,C)** Extracellular melanin in fungal extracts was black-to-light brown in color depending on the concentration and extracellular melanincontent from WT and *StRab4i* cultures was quantified in the presence (shaking) and absence (static) of shaking. Asterisks indicate statistical significance, as determined by two-tailed student’s *t*-test with three independent replicates (^**^p < 0.01). Error bars represent the standard deviation of three replicates.

The appressorium utilizes melanin to generate sufficient turgor pressure for penetrating the leaf epidermal cell layer (Ma et al., 2017). Melanin is produced intracellularly but is also a critical component of the fungal cell wall that can enter the extracellular space if the cell wall is compromised (Spanu, 1998; Ma et al., 2017). To determine whether *StRAB4* plays a role in melanin production, we compared the melanin content of the WT and *StRab4i* strains. All *S. turcica* strains were cultured in liquid medium with agitation for 3 to 4 days before intracellular melanin was extracted. Extracellular melanin was extracted from liquid cultures 10 days after inoculation. For extracellular melanin extraction, cultures were either agitated for 10 days (shaking) or not agitated (static), and the purpose of agitation was to release melanin from the cell wall. The intracellular melanin content was significantly higher in the WT, approximately 8-fold greater than in the *StRab4i-2* strain and 46-fold greater than in the *StRab4i-1* and-4 strains (Figure 5A). The extracellular melanin content in cultures that had been agitated for 10 days was similar among the WT, *StRab4i-2*, and *StRab4i-4* strains, whereas the *StRab4i-1* strain produced no visible melanin and less than 0.5 mg/g detectable melanin (Figure 5B). The extracellular melanin content in cultures that had not been agitated was highest in the WT, followed by *StRab4i-2*, *StRab4i-4*, and *StRab4i-1* (Figure 5C). The intracellular melanin content was higher than or similar to the extracellular content for all strains, except the *StRab4i-4* strain. Interestingly, the *StRab4i-4* strain had the lowest levels of detectable intracellular melanin and extracellular melanin under static conditions. However, the level of extracellular melanin in the *StRab4i-4* strain was similar as that of the WT under shaking conditions (Figure 5B). These findings suggest that *StRAB4* plays a positive role in intracellular melanin production and may be crucial for sequestering melanin in the cell wall of *S. turcica*.

### RNA-Seq analysis reveals a possible regulatory mechanism for *StRAB4* in *Setosphaeria turcica*

**Figure 6:**
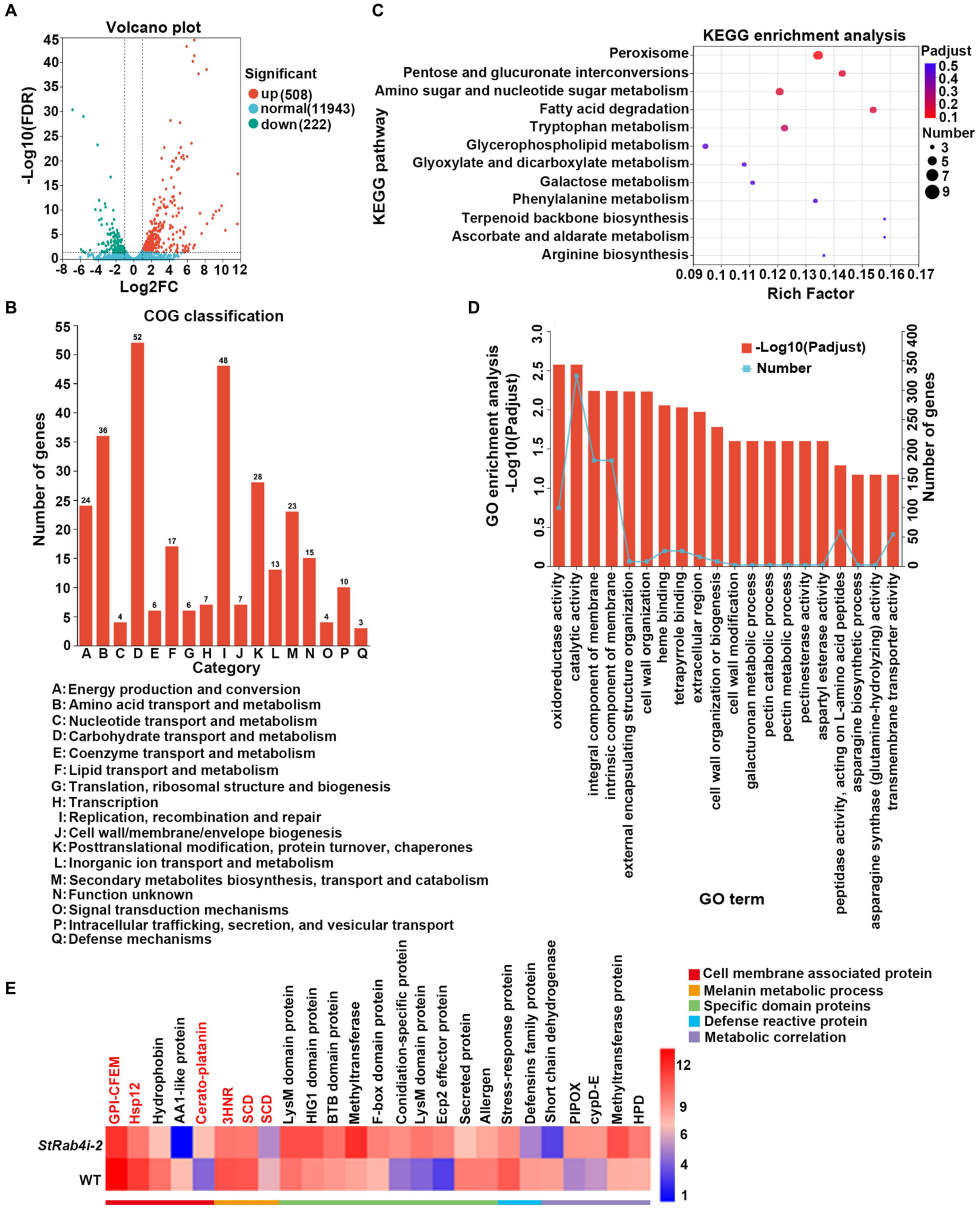
Differential gene expression and functional enrichment analysis between the WT and *StRab4i* strains. **(A)** Volcano plot depicting the differentially expressed genes (DEGs) between the WT and *StRab4i-2*. Red and green dots signify significantly up-and down-regulated genes, respectively (adjusted *p*-value ≤0.01 and log2FC >2). Blue dots signify genes that did not have significant changes in gene expression. Fold changes were calculated using fragments per kilobase million (FPKM) values. **(B)** Clusters of Orthologous Groups (COG) classification of all assembled genes. **(C)** Representation of the top 20 enriched Kyoto Encyclopedia of Genes and Genomes (KEGG) pathways among the DEGs. **(D)** Visualization of Gene Ontology (GO) terms enriched among the DEGs. **(E)** Heatmap expression of selected genes that are differentially expressed during conidia development. The color gradient from blue to red corresponds to an ascending order of expression.

**Figure 7:**
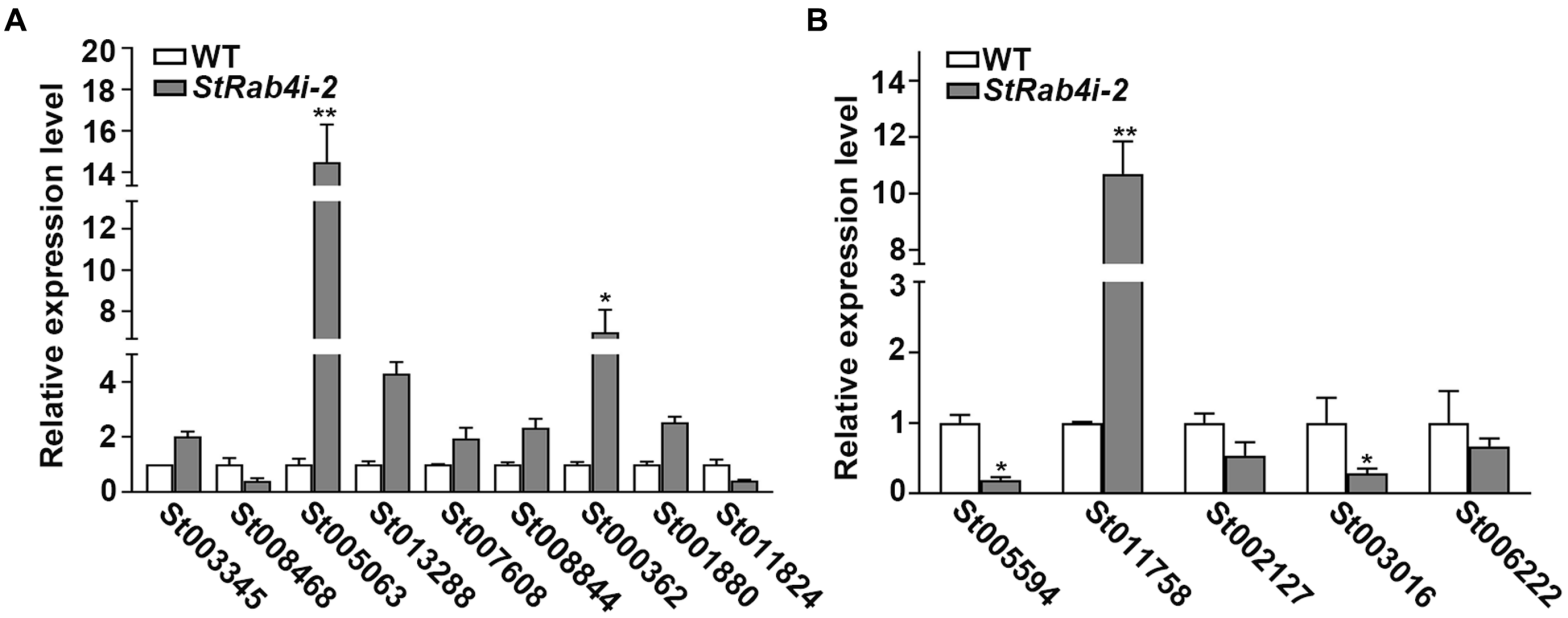
The validation of differentially expressed genes in WT and *StRab4i-2*. **(A)** Expression levels of the related genes of peroxisome. Asterisks indicate statistical significance, as determined by two-tailed student’s *t*-test with three independent replicates (^*^p < 0.05 and ^**^p < 0.01). Error bars represent the standard deviation of three replicates. **(B)** Validation levels of the differentially expressed genes (DEGs), including *HSP12* (gene ID: St005597), CPP (gene ID: St011758), *3HNR* (gene ID: St002127), GPI-CFEM (gene ID: St003016), *SCD1* (gene ID: St006222). Asterisks indicate statistical significance, as determined by two-tailed student’s *t*-test with three independent replicates (^*^p < 0.05 and ^**^p < 0.01). Error bars represent the standard deviation of three replicates.

Transcriptome data revealed that the *StRAB4* gene exhibited active expression across different developmental stages (Supplementary Figure S2). To gain a better understanding of the function played by *StRAB4* in *S. turcica,* we performed a transcriptomic comparison between the WT and *StRab4i-2* strain. In this comparison, 730 DEGs were identified with an FDR threshold of ≤0.01. Among these DEGs, 508 were upregulated and 222 were downregulated in the *StRab4i* strain (Figure 6A). COG analysis revealed that most enriched genes were involved in carbohydrate transport and metabolism, replication, recombination and repair, amino acid transport, and metabolism (Figure 6B). Furthermore, the functions of the DEGs were analyzed using the GO and KEGG databases (Figures 6C,D), and the 20 most significantly enriched terms and pathways are illustrated in Figures 6C,D. Notably, we found that peroxisome pathways (map04146) displayed the highest enrichment, revealing 7 upregulated genes (Figure 7A), while oxidoreductase activity, catalytic activity, and membrane component emerged as the most enriched GO terms.

We identified five annotated genes with potentially significant roles in the regulation of conidia morphology based on significant changes in their expression levels in the *StRab4i* strain compared to the WT (Figures 6E, 7B). These genes were annotated as GPI-anchored protein common in fungal extracellular membrane (CFEM)-domain (GPI-CFEM), heat shock protein 12 (*HSP12*), 1,3,8-trihydroxynaphthalene reductase (*3HNR*), scytalone dehydratase (*SCD1*), and cerato-platanin protein (CPP). Among these proteins, GPI-CFEM and *HSP12* are membrane related proteins (Peng et al., 2021; Wang et al., 2021), and both were found to be downregulated in the *StRab4i* strain. Conversely, *3HNR* and *SCD1* are the key synthases in melanin biosynthesis (Thompson et al., 2000; Eliahu et al., 2007; Tsuji et al., 2010), and both were also found to be downregulated in the *StRab4i* strain (Figures 6E, 7B). CPP, which possibly contribute to virulence and localize in fungal cell walls (Quarantin et al., 2016), was upregulated in the *StRab4i* strain (Figures 6E, 7B). This analysis highlights the multifaceted role of *StRab4* in *S. turcica* function and pathogenicity, providing valuable insights on fungal biology and disease progression. Moreover, these genes are interesting subjects for future studies, as their regulatory mechanisms in *S. turcica* remain poorly understood.

## Discussion

**Figure 8:**
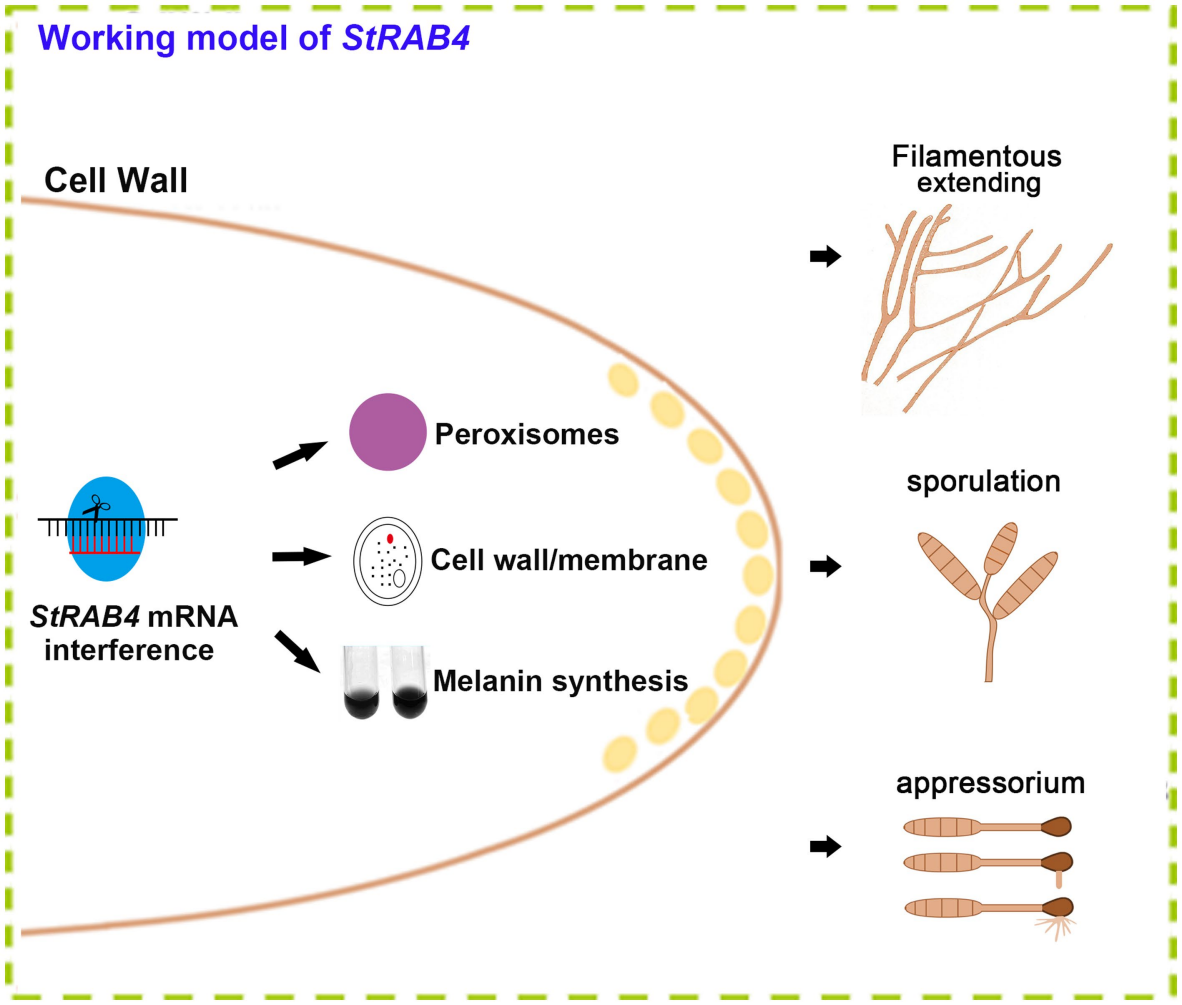
A functional model of the *StRab4i* of *S. turcica*. *StRAB4* gene plays an important role in *S. turcica*. In this study, the *StRAB4* gene functions in filamentous growth, conidial development, and pathogenicity of *S. turcica*. Further investigations revealed StRab4 is a positive regulator of cell wall integrity and melanin secretion. In the *StRab4i* mutant, functional enrichment analysis of differentially expressed genes (DEGs) highlighted primary enrichments in peroxisome pathways, cell wall organization processes, membrane components, and melanin metabolic processes.

The Rab family plays a crucial role in mycelial polar growth, a process that is vital for developing different structures such as various types of conidia, appressoria, and invasive filaments (Riquelme et al., 2011). Rab proteins act as molecular switches, facilitating the transport of vesicles between organelles in eukaryotic cells and governing the transport and distribution of proteins both inside and outside of cells. Additionally, they are integral to fungal growth, development, and pathogenesis (Zheng et al., 2015). Within the Rab family, Rab4 plays a significant role in regulating endocytosis and vesicle transport, which are essential for processes such as cell polarity, signal transduction, and pathogenic infection. *StRAB4* plays a crucial role in the development of infectious structures since it is expressed actively during all stages of growth (Supplementary Figure S2). The activity of *StRAB4* varies throughout several developmental phases, suggesting that *StRAB4* may serve diverse functions at these periods. This investigation also confirmed that this gene is required for conidial development. We highlight the essential role of *StRAB4* in the normal development of multiple infection structures used by *S. turcica* to penetrate and colonize maize leaves (Figure 8). Silencing of *StRAB4* resulted in abnormalities in conidial morphology, characterized by reduced septa, enlarged conidial ends, and disrupted appressorium-mediated penetration (Figures 1–3). The degree of silencing, as observed in *StRab4i-1*, *-2*, *-4* strains (Supplementary Figure S1), exceeded 50%, whereas the silencing efficiency in the *StRab4i-S* strain was approximately 35%. Transcriptomic analysis revealed that *StRAB4* expression did not differ significantly. One potential explanation is that gene silencing is limited to the specific segment corresponding to the gene’s fragment used in the RNAi vector, resulting in an incomplete transcript. However, the other part of the mRNA of *StRAB4* was still remaining and could be detected in the transcriptomic analysis. But for mutant identification, we used RT-qPCR with the specific primers of the silenced fragment, so the result was more accurate than the transcriptome analysis. Moreover, the mutants displayed varying phenotypes, with some even displaying contrasting features such as conidiation and hyphal growth. This variability is an inherent limitation of RNAi technology, which is not foolproof (Salame et al., 2011). RNAi technology may not always achieve absolute specificity, and it can trigger secondary effects, leading to variable silencing efficiencies and diverse phenotypes. The first thing we did was try gene knockout. The best experimental technique for changing biological genetic material is gene knockout technology. But this method has its drawbacks as well. For example, it is quite easy to induce the experimental object to die following the knockout of some critical genes, making it impossible to research these genes. Studies have found that some members of the Ras family are necessary for fungal growth, and deletion is a lethal mutation (Park et al., 2006). The RNAi method was selected in this study to investigate gene function because of the conservative nature of *RAB4* (Stenmark and Olkkonen, 2001). These observed phenomena suggest that during spore morphogenesis, the cytoskeleton becomes disordered, resulting in abnormal polar growth. Consequently, the formation and germination of spores with normal shapes are hindered, and this process may involve the participation of genes related to cell membrane component synthesis, cell wall reconstruction, and other metabolic processes. RNA-seq data revealed the primary enrichment of peroxisome pathways in KEGG pathway analysis (Figure 6C), while GO enrichment analysis revealed the enrichment of membrane components (Figure 6D). However, the precise mechanisms through which *RAB4* regulates spore development via these pathways require further investigation. Based on our experimental observations, the silence of *StRAB4* has resulted in a notable decrease in *S. turcica*’s survival capacity (Figure 2I), highlighting the significance of *StRAB4* gene function and suggesting the potential challenge of obtaining a knockout mutant. Currently, the primary limitations of *StRAB4i* mutant investigations revolve around variations in the effectiveness of gene silencing and the resulting phenotypic discrepancies across different silencing mutants. Although the RNAi silencing technique has flaws, it is the current mainstream method (Zeng et al., 2020; Liu et al., 2022). The precise subcellular location of *StRAB4* genes during various stages of development and the intricate processes by which they participate in the control of the peroxisomal pathway remain unclear. It is feasible to knockout or overexpress some genes in *S. turcica* (Liu et al., 2022), where gene knockout is aborted for *StRAB4*. In our upcoming research, we will use gene epitode-tagged techniques to manufacture the StRab4-GFP fusion protein to track the subcellular location of *StRAB4* across several developmental stages and identify its interacting proteins, so as to investigate the molecular mechanisms of *StRAB4* regulating the development and pathogenicity in *S. turcica*.

The Rab family is a crucial regulator of intracellular membrane trafficking, orchestrating the movement of materials within cells (Homma et al., 2021). Different Rab GTPases are localized at specific intracellular membranes, where they serve as key regulators of distinct steps in various membrane trafficking pathways (Stenmark and Olkkonen, 2001). This precise regulation is essential for preserving the integrity of the cell surface. In our study, the mutants exhibited noticeable differences in cell surface integrity (Figure 4). Functional enrichment analysis of DEGs revealed primary enrichments in membrane component synthesis and cell wall organization (Figure 6). We also observed a downregulation in the gene encoding common in fungal extracellular membrane (CFEM), as indicated by the RNA-seq data (Figure 6E). In *Aspergillus fumigatus*, the CFEM proteins are involved in cell wall stabilization (Vaknin et al., 2014), and this protein likely plays roles in plant pathogenicity, conidial germination, and germ tube elongation (Arya et al., 2020). In addition, the RNA-seq data revealed an upregulation in the gene encoding CPP (Figure 6E). In other fungal species, this cell wall protein which is structurally related to expansins, is associated with growth and development (Baccelli, 2015; Quarantin et al., 2016). In addition, CPP loosens cell walls and facilitates growth (Sampedro and Cosgrove, 2005), and similar to expansins, it is implicated in hyphal elongation (Baccelli, 2015) and plant infection by disrupting plant cell walls and facilitating fungal hyphae advancement or by exploiting its necrosis-inducing capability (Pazzagli et al., 2014). The role of CPP as a virulence factor has been demonstrated in pathogens such as *M. grisea* on rice and *Botrytis cinerea* on tobacco and tomato leaves (Jeong et al., 2007; Frias et al., 2011). In our study, we hypothesized that CFEM and CPP may be involved in cell wall defects and enlarged spore formation caused by *StRAB4* silencing in *S. turcica*. Another downregulated gene in our study, *HSP12*, is a membrane-associated molecular chaperone (Figure 6E). A previous study has demonstrated that *HSP12* promotes cell survival under stress conditions by folding after interacting with lipids, thereby protecting membranes in organisms such as *S. cerevisiae* (Welker et al., 2010). The combined results of our transcriptomic and phenotypic analyses of the *StRAB4* RNAi strains suggest that StRab4 plays a pivotal role in modifying the cell surface. These findings offer new insights into the complex interplay between StRab4 and cellular processes and lay a foundation for understanding its multifaceted role in fungal development and pathogenicity. Further research is necessary to reveal the underlying molecular mechanisms driving these processes.

Melanin deposits within the fungal cell wall serve diverse protective and adaptive functions in filamentous fungi. For instance, in *Aspergillus fumigatus*, melanin plays a vital role in promoting conidia adhesion to host tissues and shielding the fungus from immune system detection. It achieves this by impeding the recognition of pathogen-related molecular patterns, neutralizing reactive oxygen species produced by phagocytes, inhibiting the acidification of phagolysosomes, and prohibiting macrophage apoptosis (Chotirmall et al., 2014). Notably, we observed that some of the abnormal conidia from the *StRab4i-S* strain were colorless (Figure 2C), suggesting a deficiency in melanin within the cell wall of these conidia. Interestingly, the colorless conidia were unable to germinate, which could be attributed to the absence of the protective effects usually afforded by melanin. Our RNA-seq analysis also revealed a significant down-regulation of *St3HNR* and *StSCD1* genes in the *StRab4i* strain (Figure 6E). In phytopathogenic fungi, such as *M. grisea*, these genes participate in melanin biosynthesis (Thompson et al., 2000). However, it is unclear whether these genes play a role in *StRAB4*-mediated development and pathogenicity of *S. turcica*. These findings collectively suggest that *StRAB4* plays a role in regulating melanin biosynthesis. Given the functions of Rab4 homologs in endocytosis and vesicle transport, a more in-depth exploration of the relationship between melanin transport and vesicle dynamics is required to better understand the underlying mechanisms. This research opens the door to understanding the intricate mechanisms behind melanin biosynthesis and its role in fungal development and pathogenicity.

To explore the role of melanin, we conducted experiments to extract intracellular melanin from both the WT and *StRab4i* mutants. The results were striking, revealing that the intracellular melanin content of the WT was 46.65 mg/g, a range that was 7 to 40 times greater than that in the *StRab4i* mutants (Figure 5A). This significant difference underscores the profound influence of the *StRAB4* gene on melanin synthesis in *S. turcica*. Recent studies have proposed that fungal melanin may be synthesized and transported to the cell wall through a process used by mammalian melanosomes (Cunha et al., 2010). Given that Rab4 plays a critical role in endocytosis and vesicle transport, it is plausible that StRab4 regulates melanin transport by controlling vesicle transport. Notably, compared to the static culture, the shaking culture showed higher levels of extracellular melanin (Figures 5B,C), suggesting the porous cell wall of the *StRab4i* mutant explains the secretion of melanin, resulting in lower levels of intracellular melanin.

Melanin, as a crucial component of the cell wall, plays a pivotal role in the ability of plant pathogenic fungi to infect their hosts. Fungi create appressoria, structures that penetrate plant tissues and enable pathogens to infect their hosts. Melanin within the cell wall of these appressoria provides mechanical strength, aiding the pathogen to breach the host tissue (Eisenman and Casadevall, 2012). For instance, in *Colletotrichum kahawae*, the inhibition of melanin production reduces the turgor pressure of the appressoria, thereby diminishing the virulence of the fungus (Chen et al., 2004). Melanized appressoria are also vital for the virulence of other plant pathogens such as *M. grisea* and *Diplocarpon rosae* (Howard et al., 1996; Gachomo et al., 2010). Consequently, the decrease in pathogenicity of *StRab4i* mutants may be attributed to a reduction in melanin synthesis, underscoring how StRab4 influences the pathogenicity of *S. turcica* by regulating melanin synthesis.

Additionally, GO term and KEGG pathway enrichment analyses revealed a connection between peroxisomes and StRab4 (Figures 6, 8). Peroxisomes are ubiquitous organelles in eukaryotic cells that contain key enzymes for β-oxidation of fatty acids and detoxification of reactive oxygen species. In filamentous fungi, peroxisomes also play roles in secondary metabolism, including the biosynthesis of various compounds, and plant pathogenicity (Kimura, 2001; Saikia and Scott, 2009; Imazaki et al., 2010; Bartoszewska et al., 2011a,b; Asakura et al., 2012). These organelles also contribute to developmental processes. Disruption of peroxisome function may result in loss of cell wall integrity, thus reducing pathogenicity. Consistent with this hypothesis, deletion of *MoPEX5*, *MoPEX6*, and *MoPEX19* in *M. grisea* confers sensitivity to the cell wall-perturbing agents Congo Red and CFW. Furthermore, peroxisome-mediated cellular processes and metabolic events can enhance melanin production and maintain cell wall integrity to support *M. grisea* infection (Chen et al., 2016). In our study, this association was corroborated by the decreased sensitivity of *StRab4i* to the cell wall-perturbing agents Congo Red, CFW, and SDS (Figure 4). The involvement of peroxisomes in cell wall integrity remains an intriguing research area for future studies. In some cases, reduced pathogenicity is directly related to disrupted peroxisomal biosynthesis. For instance, when peroxisome synthesis is blocked in *M. grisea*, the mutant strain shows defects in appressoria formation and host penetration (Soundararajan et al., 2004). Peroxisomes of filamentous fungi perform a variety of functions involving both metabolism and structure. Recent studies have identified several novel and often unexpected peroxisome functions, and most of these are related to the production of antibiotics and toxins as well as developmental processes (Van der Klei and Veenhuis, 2013). Although the reasons behind the importance of peroxisomes in fungal development remain unclear, it is possible that they are involved in the production of signaling molecules, offering a promising avenue for future research (Supplementary Figure S1).

## Conclusion

This study enhances our understanding of the functions of *StRAB4* in conidial morphogenesis, cell wall integrity, and pathogenicity in *S. turcica*. These insights provide a foundation for future inquiries into the molecular mechanisms underlying these phenomena. It will be interesting to see if future studies can shed light on the role played by StRab4 in vesicle transport and how this impacts fungal morphology and pathogenesis. Further investigation in these areas could provide new avenues for managing *S. turcica* and related pathogenic fungi.

## Data availability statement

The datasets presented in this study can be found in online repositories. The names of the repository/repositories and accession number(s) can be found at: https://www.ncbi.nlm.nih.gov/, PRJNA1019470.

## Author contributions

PL: Writing – original draft. HZ: Writing – original draft. CW: Writing – original draft. FZ: Funding acquisition, Writing – review & editing. JJ: Writing – original draft. SF: Writing – original draft. XH: Writing – original draft. SS: Writing – original draft. YW: Writing – original draft. ZH: Writing – original draft. JD: Writing – original draft.

## Footnotes

^1^ http://www.ncbi.nlm.nih.gov/sra

^2^ http://ftp-private.ncbi.nlm.nih.gov
