# Supplementary material for: StRAB4 gene is required for filamentous growth, conidial development, and pathogenicity in Setosphaeria turcica
Source: Front Microbiol. 2024 Jan 8;14:1302081. doi: 10.3389/fmicb.2023.1302081 (PMC10804457; doi:10.3389/fmicb.2023.1302081)
Supplement: Supplementary file 1 [file Image_2.pdf]

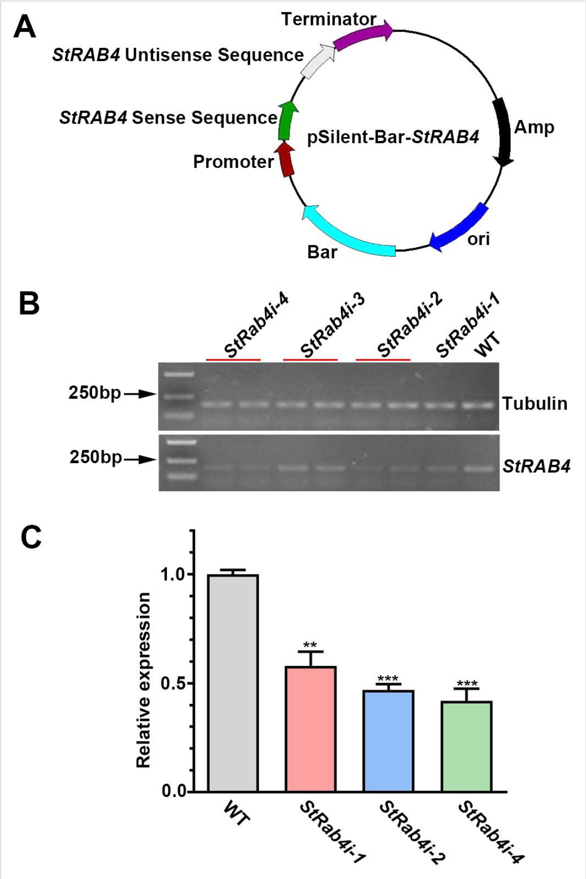

Supplementary Figure S1| Verification of the *StRab4i-1*, *StRab4i-2*, *StRab4i-3*, and *StRab4i-4* RNAi strains in *S. turcica*.

(A) Schematic representation of the pSilent-Bar-*StRAB4* construct that was used to silence *StRAB4*. **(B)** Reverse-transcription polymerase chain reaction (RT-PCR) verification of *StRAB4* gene silencing in *S. turcica*. **(C)** Quantitative PCR (qPCR) analysis to further assess the expression level of *StRAB4* in the RNAi strains. Asterisks indicate statistical significance, as determined by two-tailed Student's t-test with three independent replicates (**$P < 0.01$, ***$P < 0.001$). Error bars represent the standard deviation of three replicates.

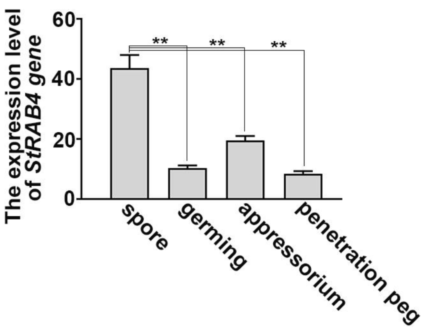

Supplementary Figure S2| The expression of the *StRAB4* gene during various stages of development. Asterisks indicate statistical significance, as determined by two-tailed Student's t-test with three independent replicates (**P < 0.01). Error bars represent the standard deviation of three replicates.
